# Supplementary material for: YerA41, a Yersinia ruckeri Bacteriophage: Determination of a Non-Sequencable DNA Bacteriophage Genome via RNA-Sequencing
Source: Viruses. 2020 Jun 5;12(6):620. doi: 10.3390/v12060620 (PMC7354516; doi:10.3390/v12060620)
Supplement: Supplementary file 1 [file viruses-12-00620-s001.pdf]

Supplementary Material

YerA41, a *Yersinia ruckeri* bacteriophage: determination of a non-sequencable DNA bacteriophage genome via RNA-sequencing.

Katarzyna Leskinen[1], Maria I. Pajunen[1], Miguel Vincente Gomez-Raya Vilanova[1], Saija Kiljunen[1], Andrew Nelson[2], Darren Smith[2] and Mikael Skurnik[1,3,*]

[1] Department of Bacteriology and Immunology, Medicum, Human Microbiome Research Program, Faculty of Medicine, University of Helsinki, Helsinki, Finland;
[2] Applied Sciences, University of Northumbria, Newcastle upon Tyne, United Kingdom
[3] Division of Clinical Microbiology, Helsinki University Hospital, HUSLAB, Helsinki, Finland
* Correspondence: mikael.skurnik@helsinki.fi; Tel.: +358-2941-26464

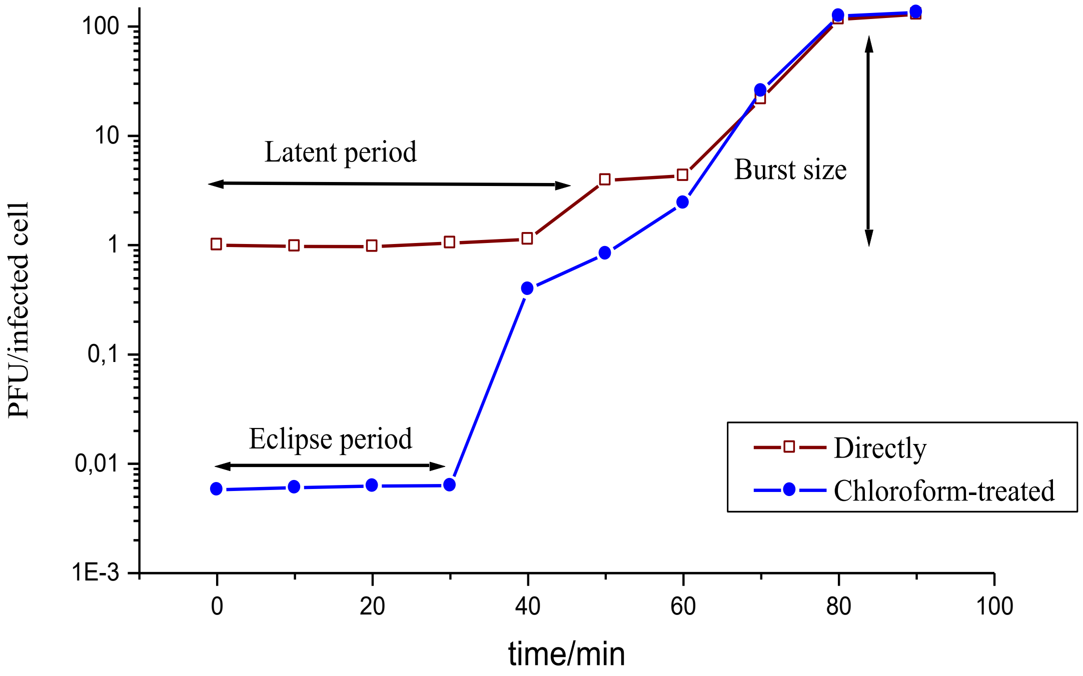

Figure S1. One-step growth curve of bacteriophage YerA41. Shown are PFU per infected cell in chloroform-treated and -untreated cultures at different time points. Each data point represents a mean of two experiments. The number of plaque forming units (PFU) in the immediately diluted 0 min time point samples was set to 1 to represent the number of infected cells in the experiment, and the PFU of all the other samples were normalized against that number. The burst size was then directly obtained from the normalized value after the rise period

Table S1. Bacterial strains used in this study (YRLBSC, Yersinia Research Laboratory Bacterial Strain Collection).

Species	Strain	O-serotype	Description	Reference(s)/Source
Shigella flexneri	784/83	-		YRLBSC
Salmonella typhimurium	ATCC13311	-		YRLBSC
Providencia rettgeri	121/84	-	patient isolate	YRLBSC
Yersinia bercovieri	3016/84	58,16	patient isolate	[1]
Yersinia bercovieri	127/84	-	patient isolate	YRLBSC
Yersinia enterocolitica	1539	-		YRLBSC
	gk132	1	patient isolate	YRLBSC
	JDE029	1		[2]
	JDE766	1,2,3		[2]
	15126/78	3		YRLBSC
	YeO3-R1	3	spontaneous rough mutant of YeO3-c	[3]
	3102/80	10	patient isolate	[4]
	3788/80	10		YRLBSC
	gc1209-79	13	patient isolate	[5]
	gc9312-78	13,18	patient isolate	[5]
	2446/84	13,7	patient isolate	YRLBSC
	421/84	13,7	patient isolate	YRLBSC
	ST5074	13a, 13b		YRLBSC
	ST5081	13a,13b		YRLBSC
	15712/83	14	patient isolate	YRLBSC
	RBC36M3	15		[6]
	IP614	15		YRLBSC
	gk2943	2	patient isolate	[7]
	gk1142	2		[7]
	gc874-77	20		[5]
	gc1223-75	20		[5]
	RBE736	21		[6]
	WI-81-50	21		[8]
	431/84	25		YRLBSC
	8425/83-ϕR1-37-R	25,26	ϕR1-37 resistant spontaneous mutant of strain 8425/83	YRLBSC
	18425/83	25,26,44	patient isolate	YRLBSC
	63/84	26,44	patient isolate	YRLBSC
	5186/84	28,50		YRLBSC
	6471/76	3	pYV+patient isolate	[4]
	gc2139-72	34		[5]
	7104/83	35,36	patient isolate	YRLBSC
	248/84	35,52	patient isolate	YRLBSC
	gc3973-76	4	patient isolate	[5]
	RBE701	4,32		[6]
	JDE701	4,32	patient isolate	[8]
	346/84	41(27), 43	patient isolate	YRLBSC
	19942/83	41(27),42 K1	patient isolate	YRLBSC
	647/83	41(27),43		YRLBSC
	626/84	41(27)42	patient isolate	YRLBSC
	9613/83	41(27)K1	patient isolate	YRLBSC
	264/85	41,43	patient isolate	YRLBSC
	477/78	5		YRLBSC
	892/82	5		YRLBSC
	14779/83	5		YRLBSC
	gk7500	5,27		[7]
	MPDE654	5,27		[9]
	3229	50		[4]
	3229-ϕR1-37-R	50	ϕR1-37 resistant spontaneous mutant of strain 3229	YRLBSC
	590/80	6	patient isolate	[4]
	341/84	6	patient isolate	YRLBSC

Species	Strain	O-serotype	Description	Reference(s)/Source
	189/80	6,30	patient isolate	[4]
	6737/80	6,30	patient isolate	[4]
	1309/80	6,31		[4]
	438/80	6,31		YRLBSC
	22848/79	7,8		YRLBSC
	605	7,8	patient isolate	YRLBSC
	1308/83	7,8		YRLBSC
	5898/83	7,8		YRLBSC
	17869/83	7,8		YRLBSC
	623/83	7,8	patient isolate	YRLBSC
	21368/83	7,8	patient isolate	YRLBSC
	21943/83	7,8	patient isolate	YRLBSC
	TAMTU	8		YRLBSC
	p310	8	patient isolate	YRLBSC
	MCH314	8	patient isolate	YRLBSC
	CDCA2635	8		[10]
	8081	8		[11]
	277/74	9	patient isolate	YRLBSC
	Ruokola/71	9	patient isolate	[4]
	4367/83	K1 nt		YRLBSC
	5653/80	na		YRLBSC
	8533/84	na	patient isolate	YRLBSC
	10/84	nt	patient isolate	YRLBSC
	gc2138-72	nt	patient isolate	[5]
	659/83	nt K1	patient isolate	YRLBSC
	15240/84	nt	patient isolate	YRLBSC
Yersinia frederikseni	146/85	-		YRLBSC
	12733/85	-		YRLBSC
	22714/85	-		YRLBSC
	3400/83	16		YRLBSC
	3317/84	35	patient isolate	YRLBSC
	38/83	48		YRLBSC
	BR166/97	-		YRLBSC
Yersinia intermedia	9/85	16,21		YRLBSC
	821/84	52,54	patient isolate	YRLBSC
Yersinia kristenseni	404/81	-		YRLBSC
	3688/85	-	patient isolate	YRLBSC
	119/84	12,25	patient isolate	[4]
	404/81	16	patient isolate	YRLBSC
	317/82	16		YRLBSC
Yersinia mollareti	92/84	59(20,36,7)		[1]
Yersinia pestis	EV76			YRLBSC
	KIM D27			YRLBSC
Yersinia pseudo-tuberculosis	2812/79	1		YRLBSC
	6088	10		[12]
	R80	11		[12]
	MW86	12		[12]
	N916	13		[12]
	CN3	14		[12]
	93422	15		[12]
	Pa3606	1b		[12]
	Kuratani	1c		[12]
	lab strain	2		YRLBSC
	p28	2		YRLBSC
	lab strain	2a		YRLBSC
	208	2a		[12]
	1779	2b		[13]
	274	2c		[12]
	324/80	3		YRLBSC
	Marsu	3		YRLBSC
	51	4a		[12]
	Pa3422	4b		[12]

Species	Strain	O-serotype	Description	Reference(s)/Source
	204	5a		[12]
	197	5b		[12]
	DD110	6		[12]
	257	7		[12]
	151	8		[12]
Yersinia similis	MW48	9		YRLBSC
	R626R	9		YRLBSC
	R708Ly	9		YRLBSC
	R708	9		[12]
Yersinia ruckeri	OMBL3			YRLBSC
	RS41		host for phage YerA41	[14]

Table S2. List of predicted protein coding genes identified in YerA41 sequence scaffolds. The phage particle associated proteins (PPAP) were identified using LC-MS/MS analysis.

geneID	Scaffold	protein length [aa]	expected function	phase of expression	PPAP
**001**	1	196	hypothetical protein	late	
**002**	1	104	hypothetical protein	late	x
**003**	1	101	hypothetical protein	late	x
**004**	1	175	hypothetical protein	late	x
**005**	1	157	hypothetical protein	late	x
**006**	1	132	hypothetical protein	late	x
**007**	1	622	Putative DNA packaging terminase	late	x
**008**	1	136	hypothetical protein	late	x
**009**	1	113	hypothetical protein	late	x
**010**	1	516	hypothetical protein	late	x
**011**	1	289	hypothetical protein	late	x
**012**	1	271	Putative prohead core protein protease	late	x
**013**	1	401	hypothetical protein	late	x
**014**	1	471	Putative capsid protein	late	x
**015**	1	285	hypothetical protein	late	
**016**	1	299	Putative sugar binding protein	late	x
**017**	1	113	hypothetical protein	late	x
**018**	1	309	hypothetical protein	late	
**019**	1	171	Putative RNA polymerase sigma factor	late	x
**020**	1	188	hypothetical protein	late	
**021**	1	199	hypothetical protein	late	
**022**	1	247	hypothetical protein	late	x
**023**	1	61	hypothetical protein	late	
**024**	1	860	Putative phage tail sheath protein	late	x
**025**	1	179	Putative tail protein	late	
**026**	1	176	hypothetical protein	late	
**027**	1	251	hypothetical protein	early	
**028**	1	1043	Putative tail family	late	x
**029**	1	221	hypothetical protein	late	
**030**	1	107	hypothetical protein	late	
**031**	1	367	Putative tail protein	late	
**032**	1	123	Putative tail protein	late	
**033**	1	723	hypothetical protein	late	

**034**	1	243	Putative tail-associated lysozyme	late	
**035**	1	95	Putative tail-associated lysozyme	late	
**036**	1	122	hypothetical protein	late	
**037**	1	551	Putative baseplate wedge	late	x
**038**	1	538	hypothetical protein	late	
**039**	1	165	hypothetical protein	late	
**040**	1	135	Putative capsid protein	late	
**041**	1	104	Putative virion structural protein	late	
**042**	1	361	Putative basplate wedge protein	late	x
**043**	1	386	Putative tail fiber protein	late	x
**044**	1	207	Phage tail fiber assembly protein	late	x
**045**	1	246	Putative tail fiber protein	late	x
**046**	1	108	hypothetical protein	late	
**047**	1	95	hypothetical protein	late	
**048**	1	113	hypothetical conserved protein	early	
**049**	1	177	Putative endolysin	late	x
**050**	1	153	hypothetical protein	late	
**051**	1	97	hypothetical protein	late	
**052**	1	169	hypothetical protein	late	x
**053**	2	230	hypothetical protein	early	
**054**	2	556	Putative DNA directed RNA polymerase, subunit	early	x
**055**	2	518	Putative DNA-directed RNA polymerase, subunit	early	x
**056**	2	924	Putative DNA-directed RNA polymerase	early	x
**057**	2	78	hypothetical protein	early	
**058**	2	335	hypothetical protein	middle	x
**059**	2	167	hypothetical protein	middle	
**060**	2	222	Putative 5'-deoxynucleotidase	middle	
**061**	2	1306	Putative DNA polymerase	middle	x
**062**	2	279	hypothetical protein	middle	
**063**	2	232	hypothetical protein	middle	
**064**	2	370	Putative UDP-GlcNAc 2-epimerase	middle	
**065**	2	257	Putative oxidoreductase	middle	
**066**	2	230	Putative SDR family oxidoreductase	middle	
**067**	2	231	Putative polysaccharide deacetylase	middle	
**068**	2	455	2-C-methyl-D-erythritol 4-phosphate cytidylyltransferase (EC 2.7.7.60)	middle	
**069**	2	393	hypothetical protein	middle	x
**070**	2	272	Putative glycerophosphodiester phosphodiesterase	middle	
**071**	2	217	hypothetical protein	middle	x
**072**	2	50	hypothetical protein	middle	
**073**	2	106	hypothetical protein	early	
**074**	2	165	hypothetical protein	middle	x
**075**	2	154	hypothetical protein	early	
**076**	2	178	hypothetical conserved protein	middle	x
**077**	2	156	hypothetical protein	middle	x
**078**	2	186	Putative lytic transglycosylase	early	x
**079**	2	97	hypothetical protein	middle	
**080**	2	106	hypothetical conserved protein	early	
**081**	2	166	hypothetical protein	early	
**082**	3	205	hypothetical protein	middle	
**083**	3	124	hypothetical protein	early	
**084**	3	177	hypothetical protein	middle	
**085**	3	60	hypothetical protein	middle	

**086**	3	304	hypothetical protein	middle	
**087**	3	116	hypothetical protein	early	
**088**	3	101	hypothetical protein	middle	
**089**	3	69	hypothetical protein	middle	
**090**	3	124	hypothetical protein	middle	
**091**	3	134	hypothetical protein	middle	
**092**	3	142	hypothetical conserved protein	middle	
**093**	3	153	hypothetical protein	middle	
**094**	3	69	hypothetical protein	middle	
**095**	3	82	hypothetical protein	middle	
**096**	3	123	hypothetical protein	early	
**097**	3	257	Putative DNA polymerase III, subunit	early	
**098**	3	62	hypothetical protein	middle	
**099**	3	204	hypothetical protein	middle	
**100**	3	179	hypothetical conserved protein	middle	
**101**	3	215	hypothetical protein	middle	
**102**	3	104	hypothetical protein	middle	
**103**	3	91	hypothetical conserved protein	middle	
**104**	3	124	hypothetical protein	middle	
**105**	3	115	hypothetical protein	middle	x
**106**	3	92	hypothetical protein	middle	
**107**	3	143	hypothetical protein	middle	
**108**	3	87	hypothetical protein	middle	
**109**	3	363	Ribonucleotide reductase	middle	
**110**	3	757	ribonucleoside-diphosphate reductase subunit alpha	middle	x
**111**	3	177	hypothetical conserved protein	middle	x
**112**	3	473	Putative ribosomal protein modification protein	middle	x
**113**	3	80	hypothetical protein	middle	
**114**	3	215	Putative dCTP deaminase	middle	
**115**	3	124	hypothetical protein	middle	
**116**	3	124	Putative RNA 2'-phosphotransferase	early	
**117**	3	216	hypothetical protein	early	
**118**	3	106	hypothetical protein	early	
**119**	3	273	hypothetical protein	early	
**120**	3	126	hypothetical protein	early	
**121**	3	58	hypothetical protein	early	
**122**	3	191	hypothetical protein	early	
**123**	3	62	hypothetical protein	early	
**124**	3	203	hypothetical protein	middle	
**125**	3	102	hypothetical protein	early	x
**126**	3	254	Putative endonuclease-like protein	early	
**127**	3	385	Putative thymidylate synthetase	middle	
**128**	3	102	hypothetical protein	middle	
**129**	3	155	hypothetical protein	middle	x
**130**	4	76	hypothetical protein	early	
**131**	4	127	hypothetical protein	early	
**132**	4	241	hypothetical protein	early	x
**133**	4	74	Putative transglycosylase	middle	
**134**	4	193	hypothetical protein	early	
**135**	4	677	Putative DNA topoisomerase	early	x
**136**	4	440	Putative DNA topoisomerase	middle	x
**137**	4	241	Putative DNA polymerase III, subunit	middle	x
**138**	4	84	hypothetical protein	middle	

**139**	4	69	hypothetical protein	middle	x
**140**	4	305	Putative structural protein	middle	
**141**	4	265	Putative dUTP diphosphatase	middle	x
**142**	4	95	hypothetical protein	middle	
**143**	4	484	Putative ATP-dependent DNA helicase	middle	x
**144**	4	54	hypothetical protein	middle	
**145**	4	359	Putative replicative helicase	middle	
**146**	4	73	hypothetical protein	middle	
**147**	4	201	hypothetical protein	middle	
**148**	4	377	hypothetical protein	middle	x
**149**	4	322	Putative exodeoxyribonuclease	middle	x
**150**	5	119	hypothetical protein	early	
**151**	5	80	hypothetical protein	middle	
**152**	5	130	hypothetical protein	middle	x
**153**	5	130	hypothetical protein	middle	
**154**	5	224	hypothetical protein	middle	x
**155**	5	171	hypothetical protein	middle	
**156**	5	106	hypothetical protein	middle	
**157**	5	142	hypothetical protein	middle	
**158**	5	80	hypothetical protein	middle	
**159**	5	155	hypothetical protein	middle	
**160**	5	190	hypothetical protein	middle	
**161**	5	251	Putative tail fiber protein	early	
**162**	5	540	Putative DNA directed RNA polymerase, subunit / Putative DNA helicase	early	
**163**	5	76	hypothetical protein	middle	
**164**	5	59	hypothetical protein	middle	
**165**	5	41	hypothetical protein	middle	
**166**	5	67	hypothetical conserved protein	middle	
**167**	5	210	Putative endonuclease	middle	x
**168**	5	94	hypothetical protein	middle	
**169**	5	141	hypothetical conserved protein	middle	
**170**	5	125	hypothetical protein	middle	
**171**	5	102	hypothetical protein	middle	
**172**	5	91	hypothetical conserved protein	middle	
**173**	5	34	hypothetical protein	middle	
**174**	5	120	Putative phage baseplate assembly protein	middle	
**175**	5	52	hypothetical protein	middle	
**176**	5	40	hypothetical protein	late	
**177**	5	82	hypothetical protein	middle	
**178**	6	203	hypothetical protein	early	
**179**	6	283	Putative exonuclease	middle	
**180**	6	1012	Putative exonuclease	middle	x
**181**	6	58	hypothetical protein	middle	x
**182**	6	173	hypothetical protein	middle	x
**183**	6	184	hypothetical conserved protein	middle	
**184**	6	138	hypothetical protein	middle	
**185**	6	201	hypothetical conserved protein	middle	x
**186**	6	68	hypothetical protein	early	
**187**	6	107	hypothetical protein	early	
**188**	6	94	hypothetical protein	early	
**189**	7	233	Putative endonuclease-like protein	early	
**190**	7	675	DNA topoisomerase	early	x

**191**	7	119	hypothetical protein	middle	
**192**	7	118	hypothetical protein	middle	
**193**	7	481	Putative helicase	early	x
**194**	8	98	hypothetical protein	early	
**195**	8	298	Putative DNA polymerase	early	
**196**	8	73	hypothetical conserved protein	early	
**197**	8	639	hypothetical protein	early	x
**198**	8	70	hypothetical protein	early	
**199**	9	637	Putative DNA ligase	early	x
**200**	9	65	hypothetical protein	-	
**201**	9	82	hypothetical protein	-	

Table S3. Transcriptional response in YerA41 infected *Y. ruckeri* cells. The lists of genes differentially expressed at 15, 30 and 60 min p.i. LogFC; log-ratio of a transcript's expression values in two different conditions. FDR; False Discovery Rate.

15 min vs 0

Gene	Function of the gene product	logFC	FDR
CSF007_0230	Promiscuous sugar phosphatase YidA%2C haloacid dehalogenase-like phosphatase family	0.86	0.000484
CSF007_0240	Outer membrane lipoprotein YidQ	-1.116	7.97×10^{-8}
CSF007_0245	16 kDa heat shock protein A	-1.37	0.000331
CSF007_0250	16 kDa heat shock protein B	-1.62	5.50×10^{-7}
CSF007_0605	Aerobic C4-dicarboxylate transporter for fumarate%2C L-malate%2C D-malate%2C succunate	-1.23	1.07×10^{-9}
CSF007_0665	Glutathione reductase	2.88	3.43×10^{-31}
CSF007_0675	Oligopeptidase A	-1.03	0.000428
CSF007_0690	Universal stress protein A	-1.20	1.55×10^{-7}
CSF007_0800	hypothetical protein	-1.12	1.77×10^{-5}
CSF007_0860	High-affinity gluconate transporter GntT	-1.38	4.39×10^{-9}
CSF007_0865	Gluconokinase	-2.12	9.37×10^{-17}
CSF007_0935	Transcriptional activator of maltose regulon%2C MalT	-1.49	7.07×10^{-14}
CSF007_0940	Maltodextrin phosphorylase	-1.31	6.96×10^{-5}
CSF007_0980	Ferrous iron-sensisng transcriptional regulator FeoC	-1.20	2.28×10^{-7}
CSF007_0985	Ferrous iron transport protein B	-1.50	6.78×10^{-11}
CSF007_0990	Ferrous iron transport protein A	-1.75	6.88×10^{-8}
CSF007_10065	Ferritin-like protein 2	1.36	1.89×10^{-7}
CSF007_1015	hypothetical protein	-0.74	0.000568
CSF007_10170	Putative membrane protein	0.99	6.75×10^{-5}
CSF007_10235	Ribosomal RNA large subunit methyltransferase A	0.84	1.69×10^{-5}
CSF007_10335	Lipid A export ATP-binding/permease protein MsbA	-0.95	3.88×10^{-5}
CSF007_10695	hypothetical protein	1.26	3.55×10^{-6}
CSF007_11010	Lysine decarboxylase%2C inducible	1.29	9.23×10^{-7}
CSF007_11035	Transcriptional repressor of PutA and PutP / Proline dehydrogenase (Proline oxidase) / Delta-1-pyrroline-5-carboxylate dehydrogenase	-1.12	9.24×10^{-6}
CSF007_11450	tRNA-Ser	-1.87	1.17×10^{-5}
CSF007_11455	hypothetical protein	-2.12	8.23×10^{-28}
CSF007_1170	Transcriptional regulator YPDSF_0092%2C LacI family	-1.06	1.61×10^{-5}
CSF007_11760	Putrescine importer	1.43	1.44×10^{-7}
CSF007_11775	Ferrichrome transport ATP-binding protein FhuC	-1.57	3.04×10^{-10}
CSF007_11780	Iron(III) dicitrate transport system permease protein FecD	-1.60	1.44×10^{-7}

CSF007_11785	Putative periplasmic substrate-binding transport protein	-1.95	3.45×10^{-15}
CSF007_11810	PTS system%2C fructose-specific IIB component-PTS system%2C fructose-specific IIC component	1.95	1.10×10^{-11}
CSF007_11820	Fructose-specific phosphocarrier protein HPr-PTS system%2C fructose-specific IIA component	1.30	3.62×10^{-5}
CSF007_11865	Mannonate dehydratase	-1.46	8.78×10^{-10}
CSF007_11875	D-mannonate oxidoreductase	-1.87	3.62×10^{-13}
CSF007_1205	hypothetical protein	-0.75	0.000372
CSF007_1210	Cyclic AMP receptor protein	-1.32	5.98×10^{-8}
CSF007_12280	Uncharacterized protein YfaZ precursor	0.98	9.55×10^{-6}
CSF007_12285	Catalase	4.10	3.26×10^{-59}
CSF007_12375	NADH-ubiquinone oxidoreductase chain E	-0.86	0.00015
CSF007_12380	NADH-ubiquinone oxidoreductase chain C-NADH-ubiquinone oxidoreductase chain D	-1.01	5.05×10^{-5}
CSF007_12385	NADH-ubiquinone oxidoreductase chain B	-0.85	0.000486
CSF007_12390	NADH ubiquinone oxidoreductase chain A	-0.90	7.72×10^{-5}
CSF007_12450	Ascorbate-specific PTS system%2C EIIA component	-3.07	6.74×10^{-23}
CSF007_12455	Putative sugar phosphotransferase component II B	-3.21	1.62×10^{-22}
CSF007_12460	membrane protein	-2.21	3.34×10^{-18}
CSF007_12505	Histidine ABC transporter%2C histidine-binding periplasmic protein precursor HisJ	-1.23	0.000314
CSF007_12510	hypothetical protein	-1.37	1.65×10^{-5}
CSF007_12625	3-oxoacyl-[acyl-carrier-protein] synthase%2C KASI	-0.89	0.000999
CSF007_12645	Putative membrane protein YfcA	0.80	0.00062
CSF007_12680	3-ketoacyl-CoA thiolase	-1.24	1.82×10^{-7}
CSF007_12840	Nucleoside permease NupC	-0.94	1.43×10^{-5}
CSF007_12845	tRNA-Lys	1.17	0.000997
CSF007_12920	hypothetical protein	0.88	0.00014
CSF007_12965	Sialic acid transporter (permease) NanT	-1.92	2.90×10^{-12}
CSF007_12985	N-acetylmannosamine-6-phosphate 2-epimerase	-0.95	0.000242
CSF007_13080	NADP-dependent malic enzyme	-1.12	9.98×10^{-8}
CSF007_1330	tRNA 5-methylaminomethyl-2-thiouridine synthase TusB	0.86	0.000399
CSF007_13380	hypothetical protein	-1.16	1.44×10^{-6}
CSF007_13405	Inosine-5-monophosphate dehydrogenase	0.91	0.000343
CSF007_13705	Sigma factor RpoE regulatory protein RseC	1.25	2.39×10^{-6}
CSF007_13710	Sigma factor RpoE negative regulatory protein RseB precursor	1.01	6.13×10^{-6}
CSF007_13720	RNA polymerase sigma factor RpoE	0.98	0.000806
CSF007_13750	Pyruvate formate-lyase	1.13	5.01×10^{-5}
CSF007_13880	Phosphosugar isomerase/binding protein	-1.01	2.19×10^{-6}
CSF007_13885	D-ribulokinase	-0.94	6.94×10^{-7}
CSF007_13895	Ribose ABC transport system%2C permease protein RbsC	-1.48	1.29×10^{-12}
CSF007_13900	Ribose/xylose/arabinose/galactoside ABC-type transport system ATP-binding protein	-2.08	2.79×10^{-28}
CSF007_13905	hypothetical protein	-3.28	2.14×10^{-13}
CSF007_13910	Ribose/xylose/arabinose/galactoside ABC-type transport system periplasmic sugar binding protein	-2.48	1.26×10^{-19}
CSF007_13970	Acid shock protein 2 precursor	-1.40	4.10×10^{-7}
CSF007_14045	hypothetical tRNA/rRNA methyltransferase yfiF	0.88	0.00058
CSF007_14050	hypothetical protein	3.31	1.55×10^{-34}
CSF007_14055	Thioredoxin 2	4.53	2.39×10^{-54}
CSF007_14060	hypothetical protein	3.64	2.92×10^{-48}
CSF007_14065	Protein acetyltransferase	2.30	5.60×10^{-23}
CSF007_14315	Hypothetical protein YqcC clustered with tRNA pseudouridine synthase C	0.82	7.39×10^{-5}

CSF007_14500	Galactose operon repressor%2C GalR-LacI family of transcriptional regulators	-0.92	1.31×10^{-6}
CSF007_14700	Predicted hemolysin III like membrane protein	-0.80	0.000572
CSF007_14715	Glycine cleavage system H protein	-0.81	0.000445
CSF007_14720	Aminomethyltransferase (glycine cleavage system T protein)	-1.11	3.59×10^{-9}
CSF007_14815	hypothetical protein	2.02	5.84×10^{-7}
CSF007_14820	hypothetical protein	3.31	1.76×10^{-11}
CSF007_15070	hypothetical protein	-0.92	1.30×10^{-6}
CSF007_15200	Amide synthase component of siderophore synthetase	2.78	6.43×10^{-21}
CSF007_15205	2%2C3-dihydro-2%2C3-dihydroxybenzoate dehydrogenase of siderophore biosynthesis	2.16	1.07×10^{-14}
CSF007_15210	Isochorismatase of siderophore biosynthesis	2.07	2.68×10^{-13}
CSF007_15255	Enterobactin synthetase component F%2C serine activating enzyme	1.52	6.32×10^{-9}
CSF007_15260	Polymyxin synthetase PmxB	1.82	9.44×10^{-19}
CSF007_15265	Enterobactin esterase	1.30	1.40×10^{-7}
CSF007_15270	Ferrichrome-iron receptor	1.10	5.01×10^{-5}
CSF007_15345	Biopolymer transport protein ExbD/TolR	-1.10	0.000176
CSF007_15665	23S rRNA (guanine-N-2-)-methyltransferase rlmG	0.84	0.000204
CSF007_15715	hypothetical protein	-2.64	2.61×10^{-10}
CSF007_15720	Hexuronate transporter	-2.38	7.15×10^{-28}
CSF007_15765	Inner membrane protein YqjF	0.95	5.66×10^{-6}
CSF007_1600	Acetyl-coenzyme A synthetase	-0.93	1.66×10^{-6}
CSF007_16000	hypothetical protein	-1.42	2.65×10^{-6}
CSF007_16005	Trehalose-6-phosphate hydrolase	-2.46	3.37×10^{-14}
CSF007_16010	PTS system%2C trehalose-specific IIB component-PTS system%2C trehalose-specific IIC component	-3.39	3.53×10^{-41}
CSF007_16315	Maltose operon periplasmic protein MalM	-1.51	6.85×10^{-6}
CSF007_16325	Maltose/maltodextrin transport ATP-binding protein MalK	-1.81	6.80×10^{-6}
CSF007_16340	Maltose/maltodextrin ABC transporter%2C substrate binding periplasmic protein MalE	-1.68	5.91×10^{-8}
CSF007_16355	PsiE protein	1.32	6.05×10^{-10}
CSF007_16360	Glucose-6-phosphate isomerase	2.24	6.00×10^{-22}
CSF007_16530	Isocitrate lyase	-0.69	0.000926
CSF007_17470	Soluble pyridine nucleotide transhydrogenase	-1.14	1.89×10^{-6}
CSF007_17480	Peroxiredoxin family protein/glutaredoxin	6.95	4.94×10^{-128}
CSF007_17485	Dihydrolipoamide dehydrogenase	7.07	1.97×10^{-146}
CSF007_17495	hypothetical protein	2.06	1.51×10^{-26}
CSF007_17500	hypothetical protein	1.79	5.04×10^{-20}
CSF007_1760	Aspartate ammonia-lyase	-1.01	6.88×10^{-5}
CSF007_17650	Glycerol uptake facilitator protein	-2.39	1.55×10^{-28}
CSF007_17655	Glycerol kinase	-2.56	1.20×10^{-19}
CSF007_17695	6-phosphofructokinase	1.21	1.03×10^{-6}
CSF007_17975	Glutamine synthetase type I	-1.09	0.00014
CSF007_17985	Nitrogen regulation protein NR(I)	-0.76	0.000579
CSF007_18075	Ribose ABC transport system%2C periplasmic ribose-binding protein RbsB	-1.41	1.96×10^{-11}
CSF007_18080	Ribose ABC transport system%2C permease protein RbsC	-0.72	0.000851
CSF007_18085	Ribose ABC transport system%2C ATP-binding protein RbsA	-1.51	7.72×10^{-10}
CSF007_18090	Ribose ABC transport system%2C high affinity permease RbsD	-1.66	1.10×10^{-11}
CSF007_1900	tRNA-Gly	0.81	7.70×10^{-5}
CSF007_3355	Aconitate hydratase 2	-1.69	3.40×10^{-12}
CSF007_5505	hypothetical protein	-0.78	1.77×10^{-5}
CSF007_5525	Glutamate Aspartate periplasmic binding protein precursor GltI	-1.56	7.31×10^{-11}
CSF007_5790	Citrate synthase (si)	-3.23	8.51×10^{-22}

CSF007_5795	Succinate dehydrogenase cytochrome b-556 subunit	-2.64	4.76×10^{-26}
CSF007_5800	Succinate dehydrogenase hydrophobic membrane anchor protein	-2.49	4.39×10^{-19}
CSF007_5805	Succinate dehydrogenase flavoprotein subunit	-2.65	2.01×10^{-22}
CSF007_5810	Succinate dehydrogenase iron-sulfur protein	-2.47	1.33×10^{-16}
CSF007_5815	2-oxoglutarate dehydrogenase E1 component	-1.80	2.19×10^{-13}
CSF007_5820	Dihydrolipoamide succinyltransferase component (E2) of 2-oxoglutarate dehydrogenase complex	-1.41	7.97×10^{-8}
CSF007_5825	Succinyl-CoA ligase [ADP-forming] beta chain	-1.60	7.19×10^{-8}
CSF007_5830	Succinyl-CoA ligase [ADP-forming] alpha chain	-1.63	1.56×10^{-9}
CSF007_5840	Cytochrome d ubiquinol oxidase subunit I	1.21	1.89×10^{-6}
CSF007_5845	Cytochrome d ubiquinol oxidase subunit II	1.14	1.98×10^{-6}
CSF007_5895	tRNA-Lys	1.27	0.000561
CSF007_5900	tRNA-Lys	0.96	5.80×10^{-7}
CSF007_5905	tRNA-Lys	1.00	5.13×10^{-8}
CSF007_5965	Galactose-1-phosphate uridylyltransferase	-1.33	7.03×10^{-8}
CSF007_5970	UDP-N-acetylglucosamine 4-epimerase-UDP-glucose 4-epimerase	-1.48	2.09×10^{-10}
CSF007_6300	Non-specific DNA-binding protein Dps / Iron-binding ferritin-like antioxidant protein / Ferroxidase	4.39	4.13×10^{-66}
CSF007_6370	Possible MFS Superfamily transporter precursor	1.04	4.09×10^{-5}
CSF007_6390	hypothetical protein	-1.65	4.00×10^{-5}
CSF007_6395	Galactose/methyl galactoside ABC transport system%2C D-galactose-binding periplasmic protein MglB	-2.08	2.72×10^{-14}
CSF007_6400	Galactose/methyl galactoside ABC transport system%2C ATP-binding protein MglA	-1.13	1.92×10^{-7}
CSF007_6500	6-phosphogluconate dehydrogenase%2C decarboxylating	1.82	1.02×10^{-13}
CSF007_6595	L-serine dehydratase%2C beta subunit-L-serine dehydratase%2C alpha subunit	-1.06	0.000227
CSF007_6600	Serine transporter	-1.30	3.63×10^{-7}
CSF007_6685	Glutaredoxin 1	3.96	8.92×10^{-56}
CSF007_6760	Arginine ABC transporter%2C periplasmic arginine-binding protein ArtI	0.95	8.89×10^{-7}
CSF007_6765	Arginine ABC transporter%2C ATP-binding protein ArtP	1.05	9.24×10^{-6}
CSF007_6870	Thioredoxin reductase	2.23	3.39×10^{-20}
CSF007_9025	Alkyl sulfatase	-0.93	2.94×10^{-6}
CSF007_9035	hypothetical protein	-1.05	9.23×10^{-7}
CSF007_9550	Putative transport protein	-1.80	4.34×10^{-12}
CSF007_9585	tRNA-Val	1.15	3.88×10^{-5}
CSF007_9590	Pyruvate kinase	1.95	7.27×10^{-15}
CSF007_9605	Sulfur acceptor protein SufE for iron-sulfur cluster assembly	0.94	0.000484
CSF007_9610	Cysteine desulfurase%2C SufS subfamily	0.97	4.77×10^{-5}
CSF007_9630	Iron binding protein SufA for iron-sulfur cluster assembly	0.71	0.000742
CSF007_9650	Phosphoenolpyruvate synthase	-1.82	6.56×10^{-15}
CSF007_9670	hypothetical protein	-2.85	1.01×10^{-17}
CSF007_9675	TonB-dependent hemin%2C ferrichrome receptor	-1.55	9.46×10^{-16}
CSF007_9680	Hemin transport protein HmuS	-1.06	1.77×10^{-5}
CSF007_9870	hypothetical protein	-1.83	7.14×10^{-6}

30 min vs 0

Gene ID	Function of the gene product	logFC	FDR
CSF007_0245	16 kDa heat shock protein A	-1.80	6.55×10^{-6}
CSF007_0605	Aerobic C4-dicarboxylate transporter for fumarate%2C L-malate%2C D-malate%2C succunate	-1.09	0.000456
CSF007_0675	Oligopeptidase A	-1.29	0.000435

CSF007_0690	Universal stress protein A	-1.56	5.98×10^{-6}
CSF007_0865	Gluconokinase	-1.80	7.28×10^{-6}
CSF007_0915	Aerobic glycerol-3-phosphate dehydrogenase	1.28	5.16×10^{-6}
CSF007_0935	Transcriptional activator of maltose regulon%2C MalT	-1.48	8.42×10^{-7}
CSF007_10015	putative lipoprotein	1.14	0.000789
CSF007_10150	PTS system%2C mannose-specific IIA component / PTS system%2C mannose-specific IIB component	-2.00	2.32×10^{-6}
CSF007_10155	PTS system%2C mannose-specific IIC component	-1.86	2.38×10^{-10}
CSF007_10160	PTS system%2C mannose-specific IID component	-1.58	5.83×10^{-5}
CSF007_10265	Lipid A acylation protein PagP%2C palmitoyltransferase	1.12	0.000305
CSF007_10385	Cold shock protein CspG	2.52	7.97×10^{-5}
CSF007_11035	Transcriptional repressor of PutA and PutP / Proline dehydrogenase (Proline oxidase) / Delta-1-pyrroline-5-carboxylate dehydrogenase	-2.14	5.30×10^{-14}
CSF007_11425	Beta-galactosidase	-1.18	4.19×10^{-5}
CSF007_11455	hypothetical protein	-1.57	4.48×10^{-5}
CSF007_1165	Putative membrane receptor protein	-1.42	2.99×10^{-7}
CSF007_11760	Putrescine importer	1.71	4.96×10^{-5}
CSF007_11865	Mannonate dehydratase	-2.13	6.04×10^{-12}
CSF007_11875	D-mannonate oxidoreductase	-2.57	4.08×10^{-14}
CSF007_1210	Cyclic AMP receptor protein	-1.42	1.30×10^{-5}
CSF007_12285	Catalase	1.54	9.40×10^{-9}
CSF007_12340	NADH-ubiquinone oxidoreductase chain L	-1.12	0.000547
CSF007_12345	NADH-ubiquinone oxidoreductase chain K	-1.38	9.35×10^{-5}
CSF007_12350	NADH-ubiquinone oxidoreductase chain J	-1.29	7.97×10^{-5}
CSF007_12355	NADH-ubiquinone oxidoreductase chain I	-1.22	0.00014
CSF007_12360	NADH-ubiquinone oxidoreductase chain H	-1.07	0.000101
CSF007_12365	NADH-ubiquinone oxidoreductase chain G	-1.17	0.000143
CSF007_12450	Ascorbate-specific PTS system%2C EIIA component	-3.17	6.76×10^{-10}
CSF007_12455	Putative sugar phosphotransferase component II B	-3.52	1.76×10^{-9}
CSF007_12460	membrane protein	-2.35	7.75×10^{-9}
CSF007_12810	Glucokinase	-2.03	3.32×10^{-5}
CSF007_12920	hypothetical protein	1.11	0.000155
CSF007_12965	Sialic acid transporter (permease) NanT	-2.72	4.08×10^{-14}
CSF007_13080	NADP-dependent malic enzyme	-1.68	2.82×10^{-9}
CSF007_13300	Putative heat shock protein YegD	1.37	4.65×10^{-5}
CSF007_13400	GMP synthase [glutamine-hydrolyzing]	1.29	5.70×10^{-5}
CSF007_13405	Inosine-5-monophosphate dehydrogenase	1.19	0.000169
CSF007_13880	Phosphosugar isomerase/binding protein	-1.98	6.52×10^{-9}
CSF007_13885	D-ribulokinase	-2.04	9.33×10^{-14}
CSF007_13895	Ribose ABC transport system%2C permease protein RbsC	-2.07	7.16×10^{-12}
CSF007_13900	Ribose/xylose/arabinose/galactoside ABC-type transport system ATP-binding protein	-2.06	7.97×10^{-5}
CSF007_13905	hypothetical protein	-5.24	6.87×10^{-10}
CSF007_13910	Ribose/xylose/arabinose/galactoside ABC-type transport system periplasmic sugar binding protein	-3.70	1.55×10^{-33}
CSF007_14710	Glycine dehydrogenase [decarboxylating] (glycine cleavage system P protein)	-1.40	2.51×10^{-6}
CSF007_14715	Glycine cleavage system H protein	-1.88	3.38×10^{-7}
CSF007_14720	Aminomethyltransferase (glycine cleavage system T protein)	-1.68	7.18×10^{-10}
CSF007_15470	3%2C4-dihydroxy-2-butanone 4-phosphate synthase	2.00	9.22×10^{-14}
CSF007_15695	Inner membrane protein ygjV	-1.22	0.00046
CSF007_15700	Altronate dehydratase	-1.53	1.13×10^{-5}
CSF007_15705	Altronate oxidoreductase	-1.56	2.24×10^{-6}
CSF007_15710	Uronate isomerase	-1.55	1.79×10^{-5}

CSF007_15715	hypothetical protein	-3.74	1.21×10^{-17}
CSF007_15720	Hexuronate transporter	-2.40	1.25×10^{-10}
CSF007_15880	Outer membrane stress sensor protease DegQ%2C serine protease	-1.41	7.04×10^{-5}
CSF007_16000	hypothetical protein	-1.99	6.12×10^{-6}
CSF007_16005	Trehalose-6-phosphate hydrolase	-3.86	3.31×10^{-24}
CSF007_16010	PTS system%2C trehalose-specific IIB component-PTS system%2C trehalose-specific IIC component	-3.85	1.29×10^{-27}
CSF007_16175	tRNA dihydrouridine synthase B	1.38	6.73×10^{-6}
CSF007_16180	DNA-binding protein Fis	1.28	0.00037
CSF007_16315	Maltose operon periplasmic protein MalM	-2.03	0.000431
CSF007_16320	Maltoporin (maltose/maltodextrin high-affinity receptor%2C phage lambda receptor protein)	-2.12	0.000568
CSF007_16325	Maltose/maltodextrin transport ATP-binding protein MalK	-2.93	3.65×10^{-8}
CSF007_16340	Maltose/maltodextrin ABC transporter%2C substrate binding periplasmic protein MalE	-2.03	1.45×10^{-6}
CSF007_17480	Peroxiredoxin family protein/glutaredoxin	1.12	7.36×10^{-5}
CSF007_17485	Dihydrolipoamide dehydrogenase	1.28	2.16×10^{-7}
CSF007_1760	Aspartate ammonia-lyase	-1.98	3.86×10^{-12}
CSF007_17650	Glycerol uptake facilitator protein	-2.08	5.48×10^{-11}
CSF007_17655	Glycerol kinase	-3.31	2.81×10^{-15}
CSF007_1770	Heat shock protein 60 family co-chaperone GroES	-1.22	0.000862
CSF007_17975	Glutamine synthetase type I	1.92	4.07×10^{-5}
CSF007_17980	Nitrogen regulation protein NtrB	1.44	1.74×10^{-7}
CSF007_18075	Ribose ABC transport system%2C periplasmic ribose-binding protein RbsB	-1.57	8.42×10^{-7}
CSF007_18085	Ribose ABC transport system%2C ATP-binding protein RbsA	-1.85	2.56×10^{-6}
CSF007_18090	Ribose ABC transport system%2C high affinity permease RbsD	-2.16	4.82×10^{-8}
CSF007_3355	Aconitate hydratase 2	-1.66	1.56×10^{-6}
CSF007_5505	hypothetical protein	-1.18	1.44×10^{-5}
CSF007_5790	Citrate synthase (si)	-3.11	8.36×10^{-10}
CSF007_5795	Succinate dehydrogenase cytochrome b-556 subunit	-2.04	9.68×10^{-13}
CSF007_5800	Succinate dehydrogenase hydrophobic membrane anchor protein	-2.64	1.15×10^{-10}
CSF007_5805	Succinate dehydrogenase flavoprotein subunit	-3.18	3.94×10^{-18}
CSF007_5810	Succinate dehydrogenase iron-sulfur protein	-3.06	6.67×10^{-15}
CSF007_5815	2-oxoglutarate dehydrogenase E1 component	-2.96	3.74×10^{-18}
CSF007_5820	Dihydrolipoamide succinyltransferase component (E2) of 2-oxoglutarate dehydrogenase complex	-2.87	1.10×10^{-12}
CSF007_5825	Succinyl-CoA ligase [ADP-forming] beta chain	-2.86	5.80×10^{-10}
CSF007_5830	Succinyl-CoA ligase [ADP-forming] alpha chain	-3.01	4.78×10^{-14}
CSF007_5840	Cytochrome d ubiquinol oxidase subunit I	1.49	0.000107
CSF007_5845	Cytochrome d ubiquinol oxidase subunit II	1.48	2.78×10^{-5}
CSF007_6145	ATP-dependent RNA helicase RhlE	1.44	0.000103
CSF007_6300	Non-specific DNA-binding protein Dps / Iron-binding ferritin-like antioxidant protein / Ferroxidase	1.90	5.48×10^{-11}
CSF007_6395	Galactose/methyl galactoside ABC transport system%2C D-galactose-binding periplasmic protein MglB	-2.79	1.40×10^{-17}
CSF007_6400	Galactose/methyl galactoside ABC transport system%2C ATP-binding protein MglA	-1.72	6.99×10^{-9}
CSF007_9025	Alkyl sulfatase	-1.32	1.03×10^{-5}
CSF007_9550	Putative transport protein	-1.51	2.19×10^{-6}
CSF007_9590	Pyruvate kinase	2.11	9.00×10^{-13}
CSF007_9650	Phosphoenolpyruvate synthase	-2.54	1.16×10^{-9}
CSF007_9675	TonB-dependent hemin%2C ferrichrome receptor	-1.13	0.000184
CSF007_9680	Hemin transport protein HmuS	-1.38	0.000972

60 min vs 0

Gene ID	Function of the gene product	logFC	FDR
CSF007_0245	16 kDa heat shock protein A	1.28	0.000686
CSF007_0915	Aerobic glycerol-3-phosphate dehydrogenase	1.66	3.28×10^{-9}
CSF007_11810	PTS system%2C fructose-specific IIB component-PTS system%2C fructose-specific IIC component	4.34	2.17×10^{-20}
CSF007_11815	1-phosphofructokinase	3.35	5.91×10^{-9}
CSF007_11820	Fructose-specific phosphocarrier protein HPr-PTS system%2C fructose-specific IIA component	4.38	4.38×10^{-13}
CSF007_11825	Putative Mut family protein	1.32	2.73×10^{-8}
CSF007_13975	Glutaredoxin-like protein NrdH	2.84	5.11×10^{-12}
CSF007_13980	Ribonucleotide reduction protein NrdI	3.24	4.52×10^{-19}
CSF007_13985	Ribonucleotide reductase of class Ib (aerobic)%2C alpha subunit	2.89	2.17×10^{-20}
CSF007_13990	Ribonucleotide reductase of class Ib (aerobic)%2C beta subunit	2.59	1.36×10^{-16}
CSF007_15205	2%2C3-dihydro-2%2C3-dihydroxybenzoate dehydrogenase of siderophore biosynthesis	1.03	0.000604
CSF007_15220	Isochorismate synthase of siderophore biosynthesis	1.10	0.000399
CSF007_17655	Glycerol kinase	1.56	3.18×10^{-5}
CSF007_9650	Phosphoenolpyruvate synthase	-1.98	1.77×10^{-13}
CSF007_9890	Manganese ABC transporter%2C periplasmic-binding protein SitA	0.99	0.000138

REFERENCES

1. Wauters, G.; Janssens, M.; Steigerwalt, A.D.; Brenner, D.J. Yersinia mollaretii sp. nov. and Yersinia bercovieri sp. nov., formerly called Yersinia enterocolitica biogroups 3A and 3B. *Int J Syst Bacteriol* **1988**, *38*, 424-429.
2. Schiemann, D.A.; Devenish, J.A. Relationship of HeLa cell infectivity to biochemical, serological, and virulence characteristics of *Yersinia enterocolitica*. *Infect. Immun.* **1982**, *35*, 497-506.
3. Al-Hendy, A. *Lipopolysaccharide of Yersinia enterocolitica: Molecular genetics and role in virulence. PhD thesis.* University of Turku: Turku, Finland, 1992; Vol. 91, p 112.
4. Skurnik, M. Lack of correlation between the presence of plasmids and fimbriae in *Yersinia enterocolitica* and *Yersinia pseudotuberculosis*. *J. Appl. Bact.* **1984**, *56*, 355-363.
5. Kay, B.A.; Wachsmuth, K.; Gemski, P.; Feeley, J.C.; Quan, T.J.; Brenner, D.J. Virulence and phenotypic characterization of *Yersinia enterocolitica* isolated from humans in the united states. *J. Clin. Microbiol.* **1983**, *17*, 128-138.
6. Perry, R.D.; Brubaker, R.R. Vwa^+ phenotype of *Yersinia enterocolitica*. *Infect. Immun.* **1983**, *40*, 166-171.
7. Kapperud, G.; Skarpeid, H.-J.; Solberg, R.; Bergan, T. Outer membrane proteins and plasmids in different Yersinia enterocolitica serogroups isolated from man and animals. *Acta path microbiol immunol scand Sect B* **1985**, *93*, 27-35.
8. Schiemann, D.A. Antigenic identity of *Yersinia enterocolitica* serotypes O:Tacoma and O21. *J Clin Microbiol* **1984**, *20*, 831-832.
9. Doyle, M.P.; Hugdahl, M.B.; Chang, M.T.; Beery, J.T. Serological relatedness of mouse-virulent Yersinia enterocolitica. *Infect Immun* **1982**, *37*, 1234-1240.
10. Gemski, P.; Lazere, J.R.; Casey, T. Plasmid associated with pathogenicity and calcium dependency of *Yersinia enterocolitica*. *Infect Immun* **1980**, *27*, 682-685.
11. Portnoy, D.A.; Falkow, S. Virulence-associated plasmids from *Yersinia enterocolitica* and *Yersinia pestis*. *J. Bacteriol.* **1981**, *148*, 877-883.
12. Tsubokura, M.; Aleksic, S. A simplified antigenic scheme for serotyping of *Yersinia pseudotuberculosis*: Phenotypic characterization of reference strains and preparation of O and H factor sera. *Contrib. Microb. Immunol.* **1995**, *13*, 99-105.
13. Samuelsson, K.; Lindberg, B.; Brubaker, R.R. Structure of O-specific side chains of lipopolysaccharides from *Yersinia pseudotuberculosis*. *J. Bacteriol.* **1974**, *117*, 1010-1016.

14. Stevenson, R.M.W.; Airdrie, D.W. Isolation of Yersinia ruckeri bacteriophages. *Appl Environm Microbiol* **1984**, *47*, 1201-1205.
